# Spermatozoa, acts as an external cue and alters the cargo and production of the extracellular vesicles derived from oviductal epithelial cells in vitro

**DOI:** 10.1007/s12079-022-00715-w

**Published:** 2022-12-05

**Authors:** Qurat Ul Ain Reshi, Kasun Godakumara, James Ord, Keerthie Dissanayake, Mohammad Mehedi Hasan, Aneta Andronowska, Paul Heath, Alireza Fazeli

**Affiliations:** 1 Institute of Veterinary Medicine and Animal Sciences, Estonian University of Life Sciences, Kreutzwaldi 62, 51006 Tartu, Estonia; 2 Department of Pathophysiology, Institute of Biomedicine and Translational Medicine, University of Tartu, Ravila St. 19, 50411 Tartu, Estonia; 3 Institute for Fish and Wildlife Health, University of Bern, Längassstrasse 122, 3012 Bern, Switzerland; 4 Department of Anatomy, Faculty of Medicine, University of Peradeniya, Peradeniya, Sri Lanka; 5 Institute for Women’s Health, Maternal and Fetal Medicine Department, University College London, 86-96 Chenies Mews, London, WC1N 1EH UK; 6 Institute of Animal Reproduction and Food Research, Polish Academy of Sciences, Tuwima St. 10, 10-748 Olsztyn, Poland; 7 Sheffield Institute for Translational Neuroscience SITraN, University of Sheffield, 385a Glossop Rd, Sheffield, S10 2HQ UK; 8 Academic Unit of Reproductive and Developmental Medicine, Department of Oncology and Metabolism, The Medical School, University of Sheffield, Sheffield, S10 2SF UK

**Keywords:** Extracellular vesicles, RNA, Preconception, Oviduct, Spermatozoa

## Abstract

The oviduct provides optimum physiological and biochemical milieu essential for successful fertilization, early embryo development and facilitates functional maturation of spermatozoa. A study has revealed that spermatozoa alters the gene expression in bovine oviductal epithelial cells (BOECs) remotely via bio-active particles, thus acting as a cue to the oviduct prior to their arrival. However, very little attention has been paid to the question of whether spermatozoa could alter the cargo of extracellular vesicles (EVs) derived from BOECs. Therefore, the aim of this study was to investigate the alterations in small non-coding RNAs in EVs cargo derived from BOECs when incubated with spermatozoa in contact and non-contact co-culture models. After 4 h of incubation the EVs were isolated from the conditioned media, followed by small non-coding sequencing of the BOEC derived EVs. Our results revealed that EVs from both co-culture models contained distinct cargo in form of miRNA, fragmented mRNA versus control. The pathway enrichment analysis revealed that EV miRNA from direct co-culture were involved in the biological processes associated with phagocytosis, macroautophagy, placenta development, cellular responses to TNF and FGF. The mRNA fragments also varied within the different groups and mapped to the exonic regions of the transcriptome providing vital insights regarding the changes in cellular transcriptome on the arrival of spermatozoa. The findings of this study suggest that spermatozoa, in contact as well as remotely, alter the EV cargo of female reproductive tract epithelial cells which might be playing an essential role in pre and post-fertilization events.

**Graphical abstract:**

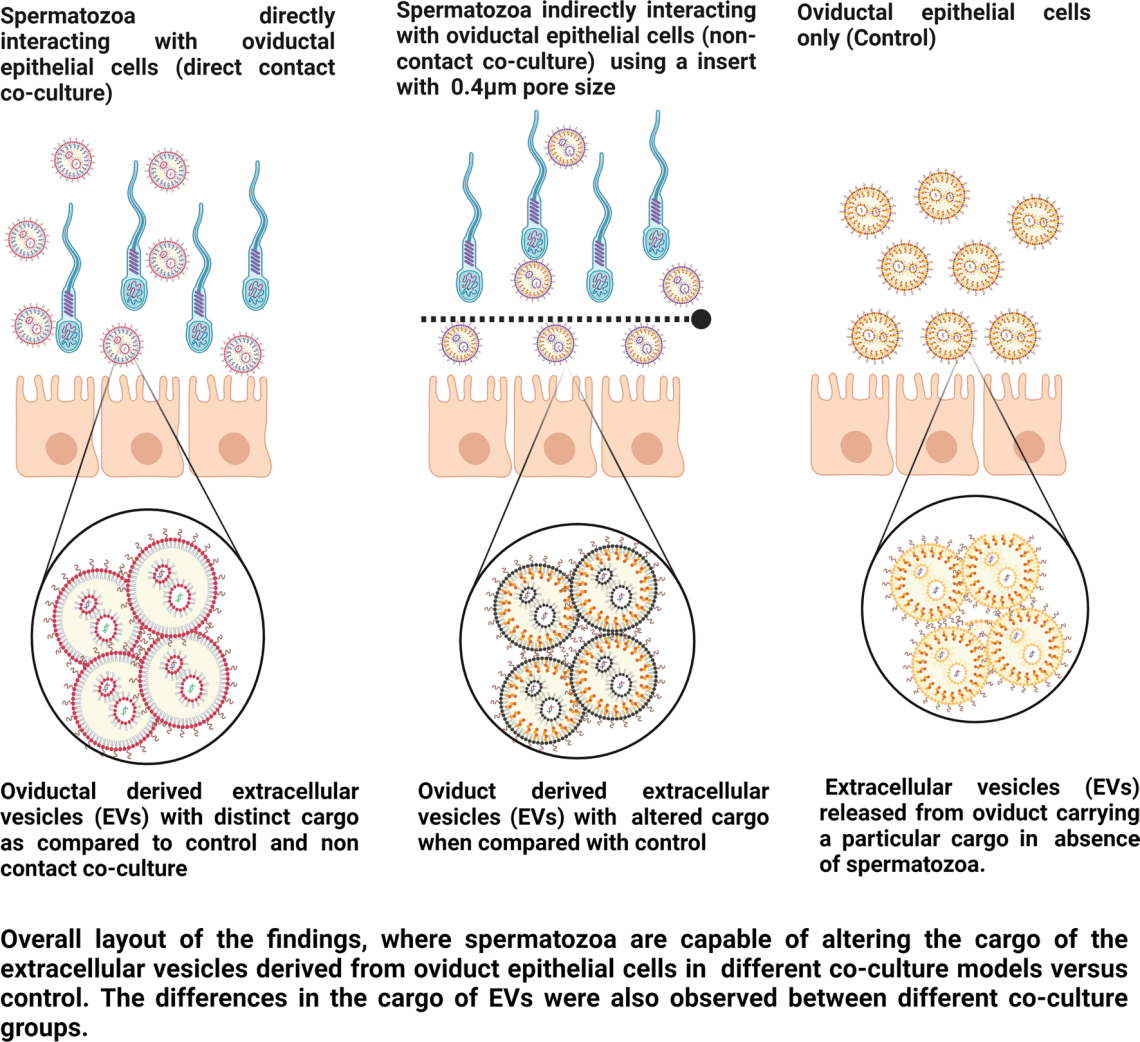

**Supplementary Information:**

The online version contains supplementary material available at 10.1007/s12079-022-00715-w.

## Introduction

In-vivo fertilization occurs in the oviduct of the female reproductive tract, which makes the oviduct a pivotal organ for the early embryo development (Dissanayake et al. 2021b; Li and Winuthayanon 2017). After the process of copulation, male gametes (spermatozoa) must travel across the female reproductive tract to reach oviducts for a successful fertilization to occur. Previous research has suggested that spermatozoa, while interacting with the oviduct, have the potential to alter the transcriptome of the oviductal epithelium (Dissanayake et al. 2021b; Fazeli et al. 2004; Kodithuwakku et al. 2007). Moreover, our recent study suggests that spermatozoa can communicate with oviductal epithelial cells remotely and alter the transcriptome of the oviductal epithelial cells (Reshi et al. 2020). This implies the existence of a cross-talk between oviduct and spermatozoa via some intercellular mediators, to maintain the optimal physiochemical milieu in the oviduct during which the fertilization may occur.

Extracellular vesicles (EVs) are one category of these intercellular communication mediators. EVs mediate intercellular communication in different reproductive events including pre and post-fertilization processes (Zhou et al. 2021). EVs are nano-sized, semi spherical biological vesicles enclosed with a lipid bilayer. They are secreted by all known types of cells and have a diameter of 30–5000 nm. EVs contain diverse cargo such as proteins, DNA, RNA and lipids (Bazzan et al. 2021; Foster et al. 2016). The three subtypes of EVs include exosomes, microvesicles and apoptotic bodies which are differentiated based on their size, function, and biogenesis (Margolis and Sadovsky 2019). EVs shuttle the cargo from one cell to another, influencing the physiology of the recipient cell. EVs are especially enriched with non-coding RNA cargo such as miRNAs, piRNAs and fragmented tRNAs which are important mediators of cell–cell communication (Artuyants et al. 2020; Chettimada et al. 2020; Gámbaro et al. 2020; O’Brien et al. 2020). Several studies have reported that the type, composition and characteristic features of the cargo within these EVs may be altered in various pathophysiological disorders (Anderson et al. 2016; Jabalee et al. 2018; Rooda et al. 2020).

Moreover, it is reported that EVs play an intricate role in enhancing the functional aspects of spermatozoa including capacitation, chemotactic motility, survival of spermatozoa in female reproductive tract and acrosome reaction in cattle (Franchi et al. 2020; Hasan et al. 2021) as well as in humans (Wang et al. 2022). Different investigations have also shown that EVs play an essential role in oocyte maturation, fertilization and embryo implantation (da Silveira et al. 2012; Machtinger et al. 2016; Sohel et al. 2013). The EVs derived from trophoblast and follicular fluid were found to alter the gene expression of the endometrium and oviductal epithelial cells respectively (Godakumara et al. 2021; Hasan et al. 2020). Research has also been carried out to examine the effects of EV-derived cargo on the various functions of the target cells to understand the role of these cargos in cell–cell communication. A recent study has reported that the presence of several EV-miRNAs released from oviductal epithelial cells in pregnant cows have the potential to initiate a dialogue between the mother and embryo, helping them in their interactions (Mazzarella et al. 2021). Even though multiple studies have revealed the importance of EVs in cellular communications, it remains unexplored whether spermatozoa affect the EV production in oviductal epithelial cells or the composition of the EV cargo produced by the oviductal cells.

Spermatozoa interact extensively with the oviduct epithelial cells and have been found to alter oviductal physiological responses (Camara Pirez et al. 2020; Rath et al. 2008). A study in dogs has found that incubation of oviduct derived EVs with oviductal epithelial cells induced maturation of oocyte especially in metaphase II phase of the cell cycle (Lange-Consiglio et al. 2017). There is a possibility that presence of thousands of spermatozoa at the fertilization site in-vivo would send biochemical signals to the oviductal epithelial cells and these cells in turn could respond by releasing EVs with distinct cargo types. Furthermore, Lange-Consiglio et al. have discovered that the cargo content of EVs recovered from canine oviducts contain miRNAs including miR-503, miR-30b and miR-375. All these miRNAs were implicated in follicular growth as well as oocyte maturation (Lange-Consiglio et al. 2017). As described above, several types of biomolecules from spermatozoa could alter the gene expression in oviductal cells (Reshi et al. 2020), however, it is not yet known whether spermatozoa could also modulate the EVs production and release from these cells as well as changes in their cargos. The expected results could provide important insights regarding the epigenetic landmarks involved in the success of fertilization events. Hence, the current investigation, was aimed to investigate the non-coding RNAs of EVs isolated from oviductal epithelial cells after incubation with spermatozoa in different co-culture models.

## Materials and methods

### Cell culture of bovine oviductal epithelial cells BOECs

The primary oviductal epithelial cells as described in a previous study (Reshi et al. 2020) were also used in these experiments. The cells were already proven to have epithelial cell markers and the cell-culture was carried out in the same way as previously. Briefly, the BOEC monolayer were cultured with DMEM/F12 media supplemented with 10% FBS, Amphotericin B (1 μl/ml) and Penicillin/Streptomycin (10 μl/ml). Once the cells attained 80% confluency the cells were washed by 1X PBS followed by pre-warmed EV depleted Sperm-TALP media prior to co-culture with spermatozoa. The cells had been passaged four times before they were co-cultured with spermatozoa.

### Spermatozoa washing and co-culture with BOECs

The co-culture experiments (oviductal epithelial cells + spermatozoa) were repeated three different times and each time ejaculates from the different bulls were used. On each day of the experiment cryopreserved semen straws were thawed for 30 s in 37 °C water bath. The constituents of three straws (225 μl of semen in each straw) were pooled and deposited onto 4 ml of 60% isosmotic Percoll^®^ solution (GE Healthcare, 17-0891-02, Sweden). The further washing of spermatozoa was carried out according to the previously described protocol (Reshi et al. 2020). Taking in consideration the number of particles contributed by the BSA (AppliChem, A1391, 0050, Germany) and its presence in the sperm-TALP media, EV depletion of the media was done. In order to minimize the number of BSA-derived particles the media was filtered through a 100 kDa Amicon^®^ Ultra-15 Centrifugal Filter Unit (R9CA01172, Ireland) (Kornilov et al. 2018). The concentration of washed spermatozoa was adjusted to 1 × 10^6^/ml and the motility was analysed in five different fields under the microscope with 40X magnification. The EV depleted Sperm-TALP media was used for co-culture of spermatozoa and oviductal epithelial cells. The spermatozoa and BOECs were incubated for 4 h and post incubation the spermatozoa motility had reduced. All the mentioned procedures were performed under aseptic conditions and the washed spermatozoa were forthwith used for co-culturing with BOECs.

### Isolation of EVs

Bovine Oviductal epithelial cells conditioned media was differentially centrifuged to get rid of cells, cell debris and apoptotic bodies. To begin with the first step of centrifugation was done at 500 × g for 10 min to pellet down all the cells. The resultant supernatant was taken ahead and centrifuged further at 2000 × g for 15 min, while maintaining the temperature of the centrifuge at 4 °C. Afterwards, the supernatant was again centrifuged at 10,000 × g for another 15 min to remove apoptotic bodies. The supernatant was transferred into Amicon filter units (10 kDa) and was concentrated until the volume reached up to 150–200 µl approximately.

Extracellular vesicles were isolated using qEVsingle size Exclusion Chromatography columns (qEVsingle/70 nm by Izon sciences, UK) and the protocol described by the Izon Science Ltd. was adopted for isolation of EVs. A recent study published has benchmarked these columns, utilizing the same protocol for isolation of EVs (Dissanayake et al. 2020). Briefly, after washing the columns with DPBS, the sample was layered on the filter and the fraction collection was started immediately (each fraction 200 μl). After the sample was absorbed by the column filter DPBS was added and the first five fractions were discarded as void volume. The fractions 6–9, containing EVs, were pooled together (total 800 μl) and concentrated using 10 KDa Amicon^®^ Ultra-15 centrifugal units.

### Nanoparticle tracking analysis (NTA)

The size profile and the concentrations of the EVs elute were determined using nanoparticle tracking analyzer-ZetaView^®^ (Particle Metrix GmbH, Germany) as described in the literature (Dissanayake et al. 2021a). The machine was standardized using a known suspension of 100 nm Polystyrene beads (Applied Microspheres B.V., Netherlands). These beads were diluted using Milli-Q water (1:250,000) and the EV samples were diluted in DPBS. The samples were measured in triplicate, and the following parameters were set in the machine while measuring: sensitivity 85, shutter speed 70, and frame rate 30 fps. Inter sample contamination was avoided by injecting DPBS in the cell before measuring another sample and the data was analysed using custom-made software.

### Transmission electron microscopy (TEM)

The morphological evaluation of EVs was carried out using a formvar/carbon coated 200 mesh grid (Agar Scientific, Stansted, UK) which was placed onto a 20 µl of concentrated EV droplet for 20 min. Afterwards, to obtain contrasted EVs, the same grid was incubated with 2% uranyl acetate (Polysciences, Warrington, PA, USA) for 5 min. The samples were air-dried and the EVs were imaged using JEM 1400 TEM (JEOL Ltd. Tokyo, Japan, with Morada TEM CCD camera, Olympus, Germany) at 80 kV. The digital images of EVs were acquired with a numeric camera (Morada TEM CCD camera, Olympus, Germany).

### Mass spectrometry

1 µg of protein was injected to an Easy-nLC 1000 system (Thermo Scientific). The sample was eluted at 250 nl/min from the trap to a 75 µm ID × 50 cm emitter-column (New Objective) packed with C18 material (3 µm, 300 Å particles, Dr Maisch). The separating gradient was 2–35% B 60 min and 40–100% B 5 min (A: 0.1% formic acid (FA), B: 80% ACN + 0.1% FA). Eluted peptides were sprayed to a Q Exactive Plus (Thermo Fisher Scientific) quadrupole-orbitrap mass spectrometer (MS) using nano-electrospray ionization at 2.4 kV (applied through liquid-junction). The MS was operated with a top-5 data-dependent acquisition strategy. Briefly, one 350–1400 m/z MS scan at a resolution setting of R = 70,000 at 200 m/z was followed by five higher-energy collisional dissociation fragmentation (normalized collision energy of 26) of 5 most intense ions (z: + 2 to + 6) at R = 17 500. MS and MS/MS ion target values were 3e6 and 5e4 with 50 ms injection time. Dynamic exclusion was limited to 40 s.

### RNA extraction from EVs

The RNA extraction from the EV elute was done using QIAzol^®^ Reagent (Qiagen, 79,306, USA) and isopropanol precipitation method. Briefly, 250 µl of guanidinium thiocyanate (QIAzol^®^ reagent) was added to 100 µl of concentrated EV elute and left at room temperature for 5 min. The contents were thoroughly homologized and 100 µl of chloroform was added and was incubated again for 5 min. Once the samples were vortexed for 15 s, they were immediately centrifuged at 12,000 × g for 15 min at 4 °C for phase separation. The aqueous phase containing RNA was transferred to a new tube and RNA was precipitated by adding 200 µl of Isopropanol and 1 µl of glycogen (UltraPure™ Glycogen, Cat. no. 10814-010, Thermo Fisher Scientific, Bleiswijk, Netherlands). The samples were incubated overnight at 4 °C and the following day samples were centrifuged for 1 h at 18,000 × g at 4 °C. The RNA pellet was washed thrice in 500 µl of 75% ethanol. The final RNA pellet was air dried, resuspended in 10 µl of nuclease free water and heated at 60 °C for 10 min. The quality of RNA was assessed with Agilent RNA 6000 Pico kit and the amount was quantified pre-library preparation using Qubit™ RNA High Sensitivity (HS) kit (Q32852, ThermoFisher Scientific) with spiked in RNA to extend the range. EV derived RNA is very different from cellular RNA and does not have the requisite ratio of ribosomal RNA to provide a RIN number when analysed with bioanalyzer. Therefore, the quality of EV RNA was not quantified using conventional methods. The amount of input RNA was determined using the range extender method described by Li et al. (2015).

### Small RNA library preparation

The small RNA libraries were prepared using a RealSeq^®^-AC miRNA Library Kit (Cat no 500-00012), following the manufacturers protocol. In order to amplify the reverse transcription product, twenty cycles of PCR were performed. The quality of the libraries was accessed using Qubit dsDNA HS Assay Kit (ThermoFisher cat Q32851) and Agilent DNA 1000 Kit.

### RNA Sequencing of oviductal epithelial cells derived EVs

The libraries were quantified by Qubit (High sensitivity dsDNA kit, protocol as per manufacturer instructions) and ran on Tapestation 2200 (D1000 kit, protocol as per manufacturer instructions). The molarity of libraries were calculated from Qubit concentration and Tapestation sizing. The Sequencing carried out on an Illumina HiSeq 2500 in rapid mode, using on-board clustering, single read cluster kit and Flow Cell, 50 cycle SBS kit. The read length was 1 × 50 bp, 6 bp single index read. Each library was run on two sequencing lanes, producing two output FASTQ files for each sample.

### Read quality control, alignment, and counting

Read trimming was performed using cutadapt v2.5 (REF) using the parameters ‘-u 1 -q 20—trim-n -m 15 -a TGGAATTCTCGGGTGCCAAGG’ to clip the first base (recommended for RealSeq-AC libraries), remove reads with average phred33 base quality lower than 20, trim N bases from the 3’ end, remove reads shorter than 15nt, and remove the adapter sequence, respectively. The two trimmed FASTQ files for each sample were then concatenated together. For subsequent analyses, we kept reads with a maximum length of 30nt.

### Read alignment to genome

Reads were aligned to the Bos taurus assembly ARS-UCD-1.2 using STAR version 2.7.9a (Dobin et al. 2013). Alignment parameters were chosen depending on the length of reads to be aligned. Reads in the 16-48nt range were aligned in EndToEnd mode using the following additional nondefault parameters:—outFilterMultimapScoreRange 0—outFilterMatchNmin 16—outFilterScoreMinOverLread 0—outFilterMatchNminOverLread 0.8—alignSJDBoverhangMin 1000—alignIntronMax 1. These parameters permitted alignments with a minimum of 16 matches while allowing the number of permitted mismatches to vary with read length and did not permit spliced alignments (no more than 20% of the read length). Reads in the 49-50nt range were aligned in EndToEnd mode with following additional nondefault parameters:—outFilterMultimapScoreRange 0,—outFilterScoreMinOverLread 0—outFilterMatchNminOverLread 0. These parameters permitted alignments with the default minimum of 10 mismatches (approx. 20% of the read length), while permitting spliced alignments. Sequence length histograms of aligned reads were derived using samtools version 1.15.1 (Danecek et al. 2021).

### Characterising RNA composition of BOEC EVs by alignments to genomic annotations

To derive a broad characterisation the small RNA landscape of BOEC EVs, alignments to various genomic features (specifically different RNA classes) were quantified from the pooled genome alignments of all samples, using a custom annotation file. Briefly, ENSEMBL gene annotations (ARS-UCD1.2.105), which include protein-coding gene coordinates in addition to several noncoding RNAs, were augmented with coordinates of piRNA clusters obtained from the piRNA cluster database (Rosenkranz 2016) and tRNA gene coordinates obtained from the genomic tRNA database (Chan and Lowe 2009). Coordinates of intron regions were obtained by subtracting exon regions from complete genes using bedtools subtract (bedools version 2.29.2, Quinlan and Hall 2010). As an appreciable proportion of miRNA genes are known to reside within introns (Steiman-Shimony et al. 2018), the intron coordinates were filtered to remove miRNA genes (included in ENSEMBL annotation), such that alignments would not be classed as ambiguous due to overlapping with both intron and miRNA annotations. Indeed, 517 out of 951 miRNA annotations were found to overlap with introns. All features in the augmented annotation (including individual introns and exons) were classed as ‘gene’ for the purpose of counting alignments at gene-level with featureCounts (from Subread version 2.0.1, Liao et al. 2014) using parameters: -t 'gene'—fracOverlap 0.6—largestOverlap—extraAttributes 'gene_biotype'. Alignments of both uniquely-mapping and multimapping reads were counted, the latter by supplying the ‘-M’ option to featureCounts. Subsequent pie charts showing percentages of alignments to different annotated features were generated using the ggplot2 package in R (Wickham 2011). As well as examining features linked to uniquely mapping and multimapping reads separately, we also examined three ranges of read length separately: (1) reads in the 16–30 nt range, known to harbour the best known classes of small noncoding RNAs (miRNA and piRNA), (2) reads in the 31–48 nt range which may comprise other small RNAs of intermediate size (including tRNA-derived small RNAs), and (3) reads in the 49–50 nt range which may have derived from transcripts longer than the maximum read length of 50 nt. Features were included in a given pie chart if they comprised at least 1% of the total alignments within a given category; features comprising < 1% were represented in the category ‘other’.

### Read counting for gene-level differential expression of mRNA

Alignments in the 16–48 nt range that overlapped with protein-coding gene annotations were counted separately for differential expression analysis given that the former likely derive from fragmented mRNA while the latter may be more likely to derive from full-length transcripts. Non-multimapping alignments were counted to either exons or introns and summarised at the gene level, such that differentially expressed genes could be identified separately from reads aligning to exons and introns. To count reads aligned to exons, we used a subset of the Bos_taurus.ARS-UCD1.2.105.gtf annotation file that had been filtered to include only protein-coding gene coordinates. To count reads aligned to introns, we derived an annotation file containing coordinates of individual introns using code from the CRIES repository (https://github.com/csglab/CRIES). In each case, featureCounts was run with parameters—fracOverlap 0.6—largestOverlap. Either ‘-t exon’ or ‘-t intron’ was specified depending on whether alignments to exons or introns were to be counted.

### miRNA alignment and counting

For alignment and quantification of miRNAs, the trimmed reads were aligned directly to *Bos taurus* mature miRNA sequences from miRbase using bowtie2/2.4.1 with default settings thus removing the possibility of alignments to immature miRNA. High quality primary alignments (SAM flag 0 × 904, MAPQ = / > 10) to each transcript were counted using samtools version 1.9. These transcript-level counts were then used as input for differential expression analysis.

### Protein-coding gene expression quantification

To examine the expression of protein-coding genes, we used the whole-genome alignments obtained using STAR and used htseq-count to count alignments of non-multimapping reads against ENSEMBL *Bos taurus* protein-coding gene annotations at the gene level, considering only alignments to exons (htseq-count parameters: ‘-t exon -i gene_id—stranded = no’). These gene-level counts were then used as input for differential expression analysis.

### Differential expression analysis (miRNA and mRNA fragments)

Differential expression (DE) analysis was carried out in R version 4.1 using the edgeR package version 3.36.0 (Chen et al. 2016; McCarthy et al. 2012; Robinson et al. 2010). Tagwise dispersion estimates were obtained based on the trended dispersions, and statistical comparisons were performed using a generalized linear model followed by likelihood ratio tests, also accounting for the experiment batch. We considered the differential expression of genes with a false discovery rate (FDR) ≤ 0.05 to be statistically significant.

Principal components were calculated using prcomp function from the Stats package and visualized using the ggplot2 package (Wickham 2016). The pheatmap package (“pheatmap”) was used for heatmap visualization with hierarchical clustering based on Euclidean distance.

### miRNA targets and functional enrichment analyses

For differentially expressed miRNAs, we obtained a list of all predicted target transcripts from miRDB (Chen and Wang 2020). These were filtered to retain only high-confidence targets (those with a target score of ≥ 90). Using the R package AnnotationDbi (Nie et al. 2009), REFSEQ transcript IDs were converted to ENSEMBL gene IDs to obtain the list of predicted miR targets at the gene level. We were thus able to identify putative high confidence miRNA targets of the miRNA identified in EVs.

To derive putative functional insight into differentially expressed protein-coding genes and predicted targets of differentially expressed miRNAs, gene set enrichment analyses (GSEA) were performed using the enricher function (for predicted targets) and gseGO function (for the ranked list of differentially expressed exonic mRNA fragments) from the clusterProfiler package (Yu et al. 2012) to identify overrepresented Gene Ontology (GO) terms. GO term annotations for *Bos taurus* genes were obtained from ENSEMBL Biomart using the biomaRt package (REF). Transcriptomic data collected from unaltered bovine embryos deposited in the National Center for Biotechnology Information (NCBI) Gene Expression Omnibus (GEO) database (https://www.ncbi.nlm.nih.gov/gds) under the accession number GSE192908 were used as the background dataset (Sidi et al. 2022).

### Mass spectrometry data analysis

Mass spectrometric raw files were processed with the MaxQuant software package (versions 1.6.15.0 and 2.0.3.0). Methionine oxidation, asparagine and glutamine deamidation and protein N-terminal acetylation were set as variable modifications, while cysteine carbamidomethylation was defined as a fixed modification. Label-free protein quantification (LFQ) was enabled with LFQ and protein minimum ratio count set to 1. Search was performed against *Bos taurus* reference proteomes, using the tryptic digestion rule. Peptide-spectrum match and protein false discovery rate (FDR) were kept below 1% using a target-decoy approach. All other parameters were default. The log transformed intensity values (not LFQ values, as it did not yield adequate number of positive hits), which are considered to correlate with the abundance of proteins, were visualized in a heatmap for 30 selected proteins based on MISEV 2018 (Théry et al. 2018) using R. The overlap of top 100 proteins reported in the Vesiclepedia (“Vesiclepedia: Home—Extracellular vesicles database,” n.d.) and Exocarta databases (Keerthikumar et al. 2016) and the proteins reported in the EVs in the current study were visualized in a Venn diagram using InteractiveVenn (Heberle et al. 2015).

### Experimental design

**Fig. 1:**
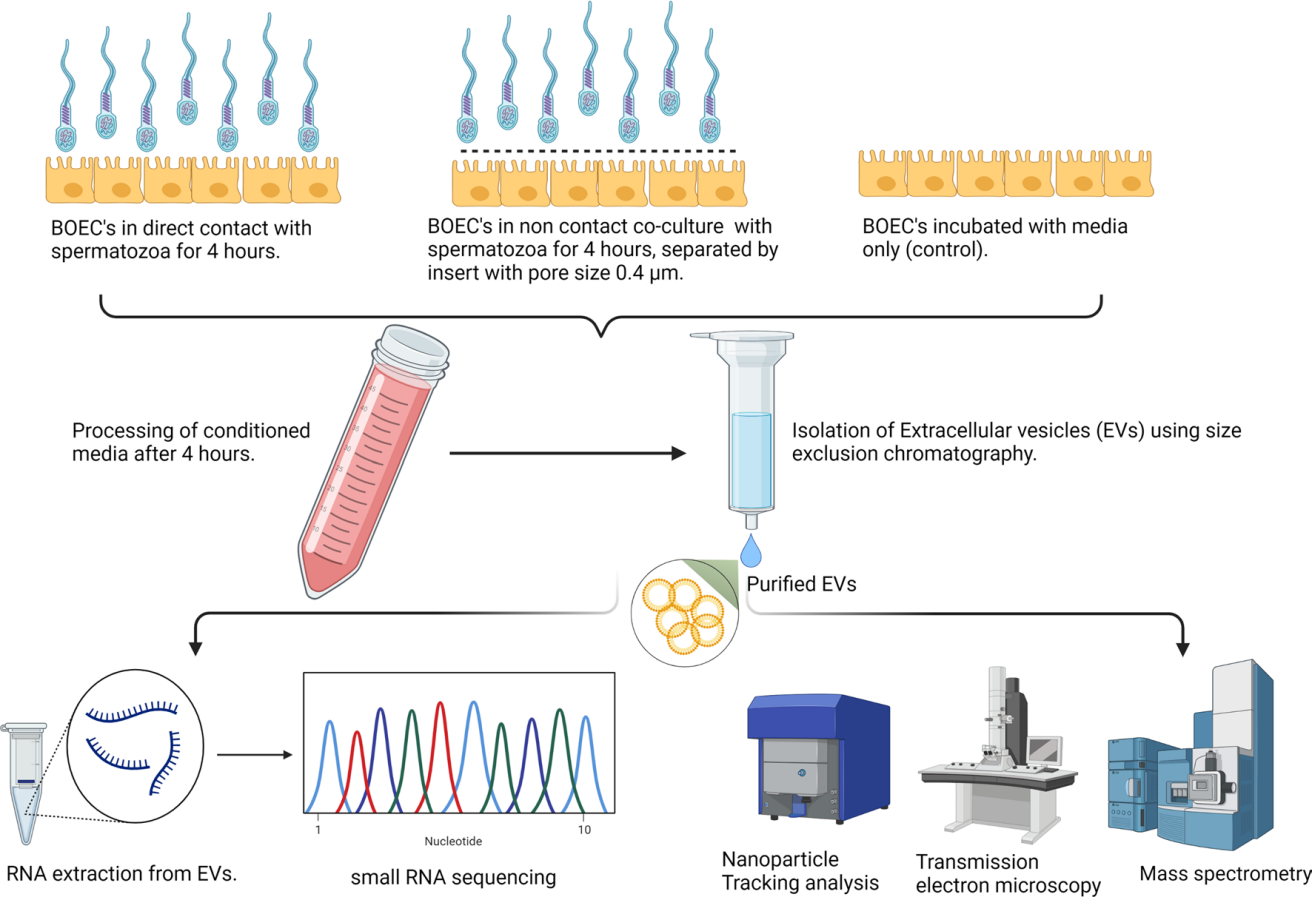
Overall layout of the experimental design used in the study. The EVs obtained from conditioned media of all the three groups were characterized by NTA, TEM and Mass spectrometry. The RNA obtained from EVs was sequenced and the comparisons within the three groups (direct contact (DC), non-contact (NC), control (C)) were made based on the changes in the cargo

During the course of experiments, the cargo of the BOECs derived EVs when incubated with spermatozoa in a contact and a non-contact co-culture model was compared with BOECs derived EVs (control). The following illustration (Fig. 1) demonstrates our experimental set up. During this experiment, the frozen thawed BOECs from three different cows were cultured separately until they attained 80% confluency. Afterwards the cells from three different oviducts were mixed and seeded in 12 well culture plates (100,000 cells/well). When the cells attained confluency, the direct contact (DC) and non-contact (NC) co-culture with spermatozoa was performed. This experiment was done three times on three different days using different aliquots of the same batch of primary cells each time. In contact co-culture model, the spermatozoa (1 × 10^6^ spermatozoa/ml) were directly added onto the BOECs where as in non-contact co-culture model an insert (Thincert cell culture insert, Greiner Bio-one GmbH, Kremsmunster, Austria) of 0.4 µm pore size was placed between spermatozoa and BOECs. Insert made of inert materials were used during experiments to avoid interference with the experiments conducted. In case of non-contact co-culture the spermatozoa was added onto the top of the insert and the spermatozoa concentration was same as that of contact co-culture (1 × 10^6^ spermatozoa/ml). On each experimental day, spermatozoa of a different bull was prepared (as mentioned earlier) and co-incubated for 4 h with bovine oviductal epithelial cells in EV depleted Sperm-TALP media. After the incubation period was over, the conditioned media was collected from all the groups separately, followed by isolation of EVs. After isolating the EVs by employing SEC the size and concentration of EV elute was determined by NTA, morphology was determined by TEM and biochemical characterization for EV markers was performed by mass spectrometry. The mass spectrometry was performed on EVs isolated from the control sample but not from the contact/non-contact co-cultures. Since the aim of mass spectrometry was to perform a qualitative analysis to show the presence of EVs in our study samples, we used single replicates from each category. i.e. 1 replicate for BOEC EV group (enriched EV elute) and 1 replicate for BOEC culture media (unpurified sample/neat). In order to analyze the cargo of EVs from each group, the RNA was extracted from the EVs followed by library preparation and small RNA sequencing.

## Results

### Characterization of EVs

**Fig. 2:**
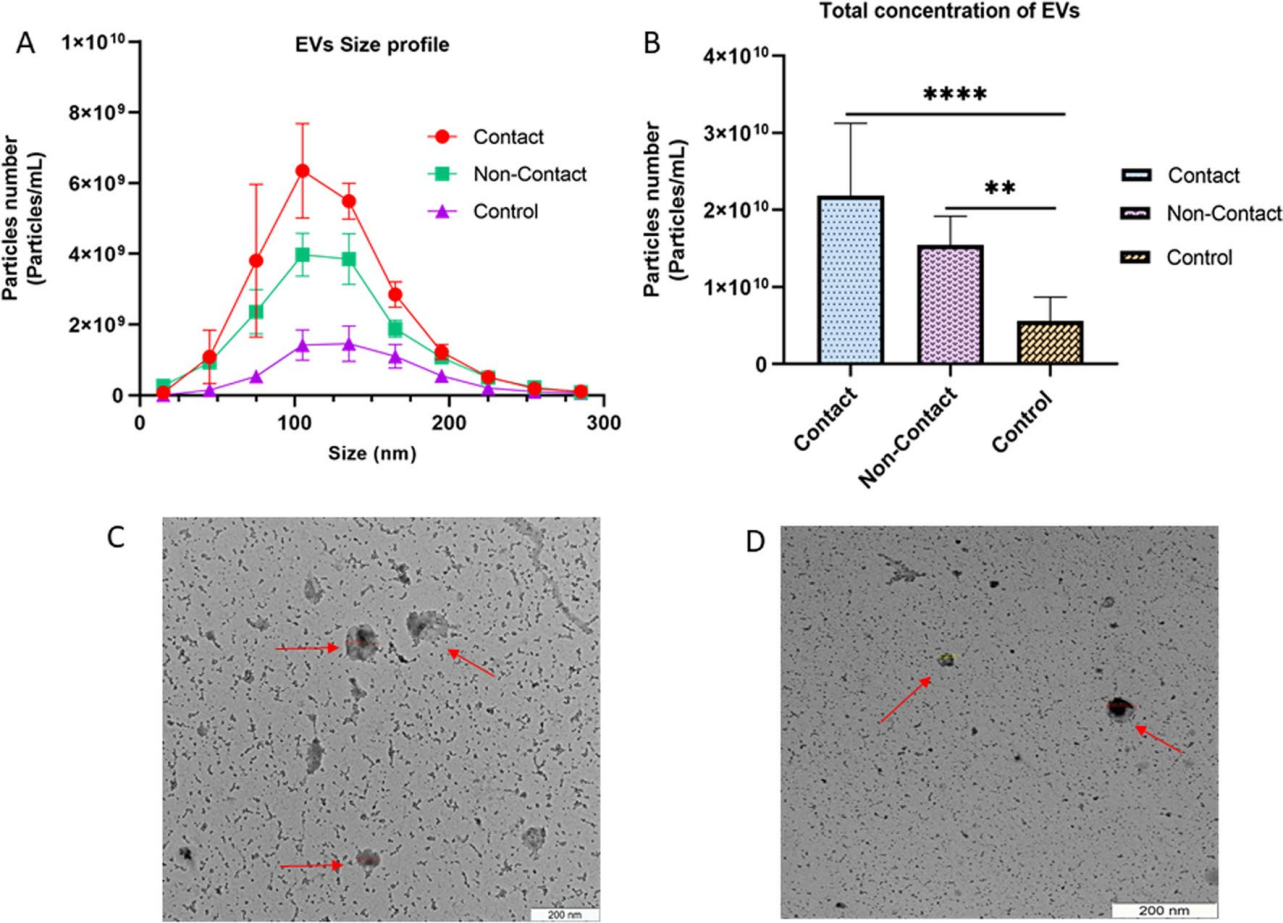
Characterization of oviductal epithelial cells derived EVs (**A**) The size profile of the EVs where most of them lie in the range of 70–105 nm which is the typical size of EVs (**B**) The total concentration of the EVs measured by NTA in all the three groups. **C** Morphological evaluation of EVs derived from contact co-culture of oviductal epithelial cells and spermatozoa. **D** Morphological evaluation of EVs derived from non-contact co-culture of oviductal epithelial cells and spermatozoa

EVs size in all the three groups was in range of 75–105 nm (Fig. 2A). The total particle concentration from contact co-culture, non-contact co-culture system and control were 2.19 × 10^10^/ml ± 5.08 × 10^9^, 1.55 × 10^11^/ml ± 2.01 × 10^9^ and 5.64 × 10^9^/ml ± 1.64 × 10^10^ (mean ± SEM; n = 3) respectively (Fig. 2B). Furthermore, the concentration of EVs in the contact (*p* < 0.0001) and non-contact co-culture system (*p* = 0.0062) were found to be statistically significant in comparison to the control. There were a higher number of EVs in the contact and non-contact co-culture groups compared to the control samples which implies that in the presence of spermatozoa, oviductal cells release more EVs.

### Transmission electron microscopy

The analysis made with Transmission electron microscopy (TEM) revealed that EVs isolated from contact (Fig. 2C) and non-contact (Fig. 2D) co-culture systems were cup-shaped which has been described as the typical shape of the EVs in the literature (Es-Haghi et al. 2019; Hasan et al. 2020).

### Mass spectrometry

**Fig. 3:**
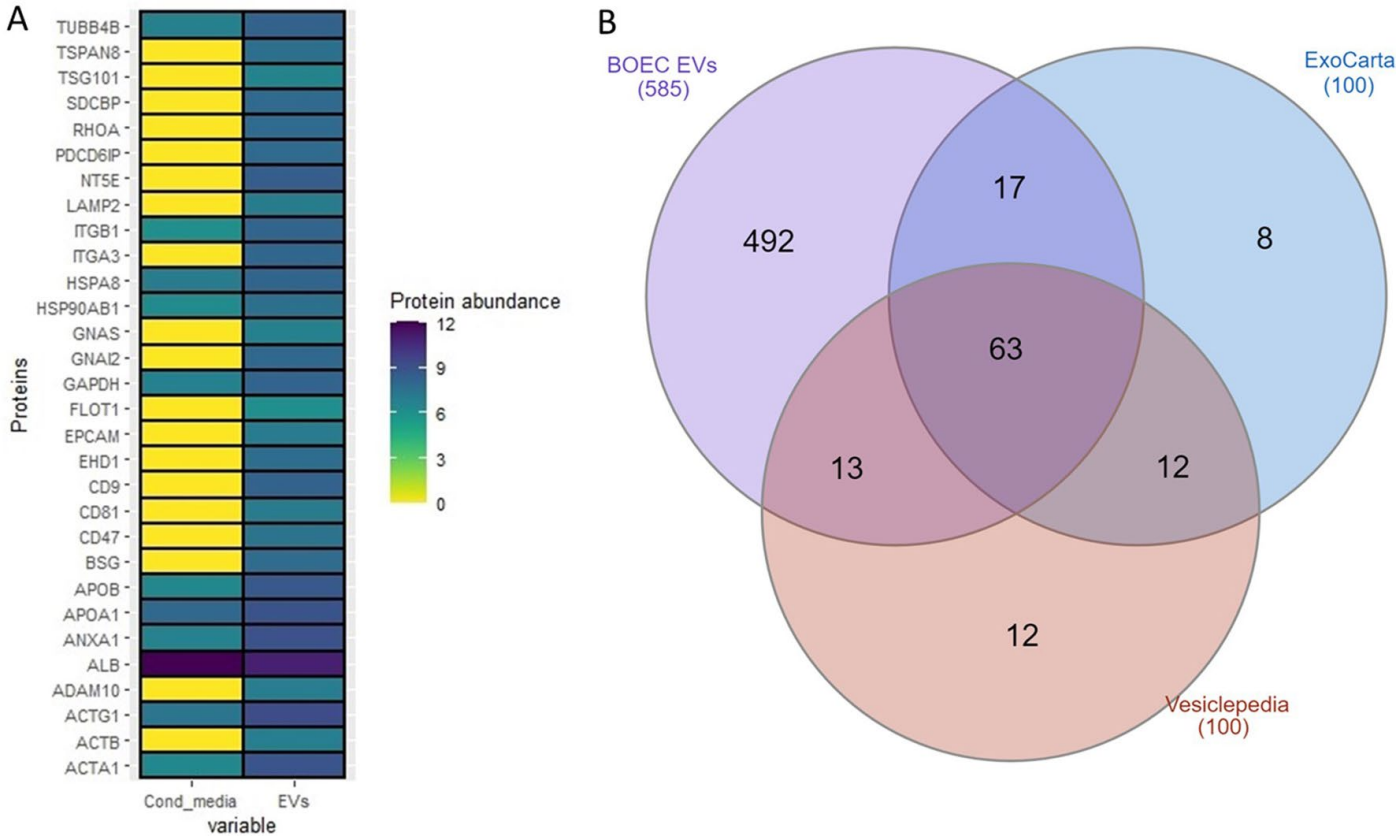
Mass spectrometry analysis of EV associated marker proteins **A** Heatmap illustrates proteins enrichment in EVs compared to BOEC conditioned media samples based on their relative abundances. The reported proteins in the heat map (30 proteins) are selected from the Vesiclepedia top 100 proteins list as the most reported proteins. **B** The overlap of proteins detected in BOEC EVs and the top 100 proteins reported in the Vesiclepedia and Exocarta databases are shown in Vendiagram. The Vendiagram was created using InteractiveVenn

The mass-spectrometry comparison between the neat/unpurified conditioned media and enriched EVs elute confirmed that the enriched fractions contained exosomal proteins and EV markers. However, it was observed that even after SEC, the expected depletion of negative protein markers such as albumin and APO A1 was not evident. However there was a drop in the intensity of albumin, and we were able to obtain the enrichment of the EVs. Overall, label-free quantification analysis of BOEC EV and BOEC conditioned media samples detected 589 proteins. Of the top 100 proteins in the Vesiclepedia protein list, 76 were reported in the BOEC EVs. The enrichment of proteins in the BOEC EVs compared to the conditioned media in selected proteins is illustrated in figure (Fig. 3A). The list of 589 proteins reported from the mass-spectrometry analysis was cross-referenced with the top 100 reported proteins lists of the public EV proteome databases Exocarta and Vesiclepedia. Out of the top 100 EV protein list in Vesiclepedia database, 76 proteins were detected in the BOEC EV protein sample, whereas from Exocarta database among top 100 EV proteins, 90 proteins were detected in the BOEC EVs sample (Fig. 3B). The complete list of proteins in BOEC neat versus enriched BOEC EVs can be found in supplementary material (Table S1). The mass spectrometry proteomics data have been deposited to the ProteomeXchange Consortium via the PRIDE (Perez-Riverol et al. 2022) partner repository with the dataset identifier PXD038106. In addition we also have added the raw values of the mass-spectrometry max-quant raw data file in the supplementary data.

### The EVs derived from oviductal epithelial carry miRNAs and fragmented mRNAs which are differentially expressed in both co-culture groups versus control

#### Small RNA composition of EVs

**Fig. 4:**
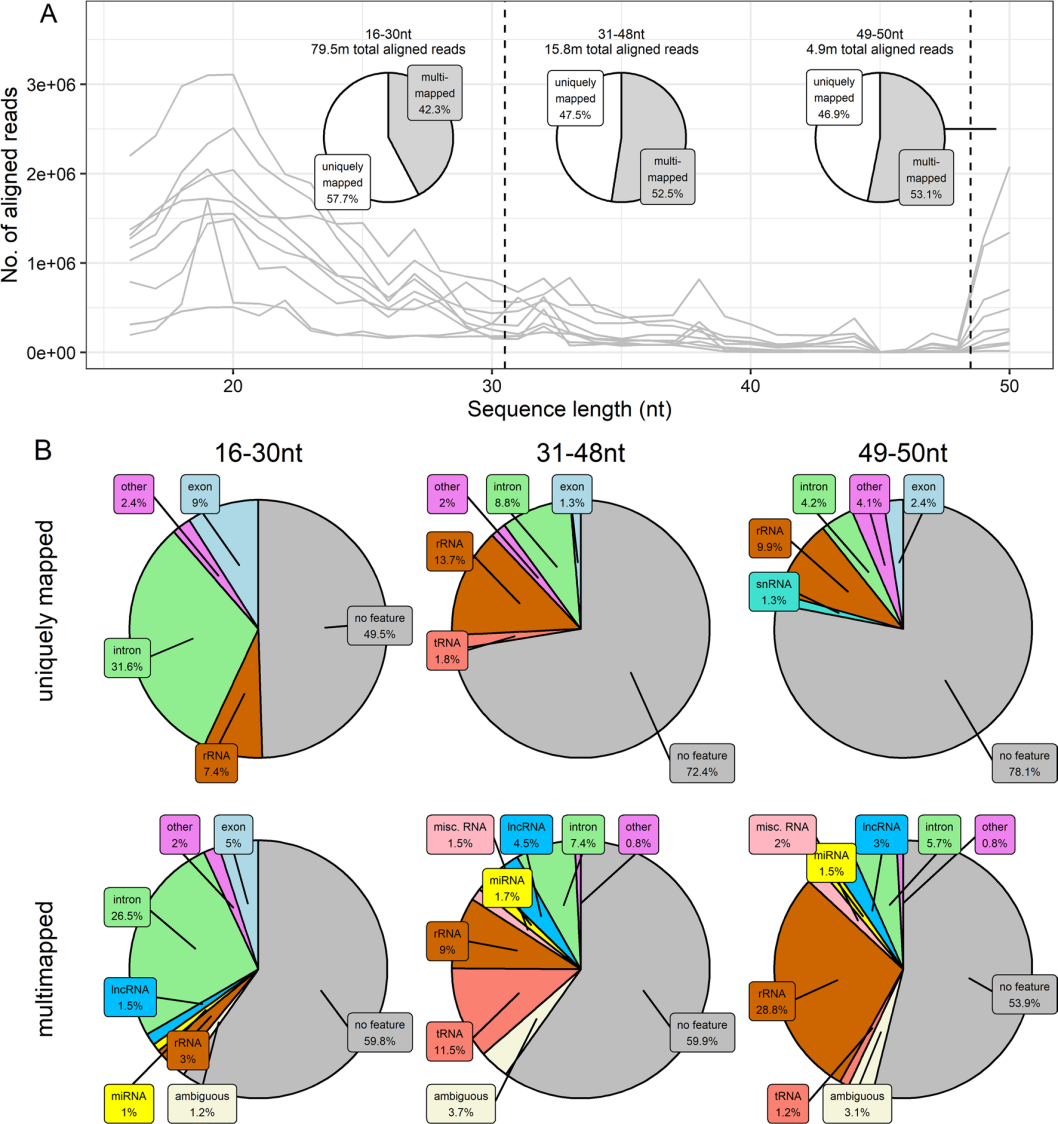
Small RNA composition of BOEC-derived EVs. **A** Numbers of trimmed reads successfully aligned to the genome (ARS-UCD1.2.105) across the range of read lengths, where each line represents reads from one sample. Dashed lines divide the three sub-ranges considered for detailed compositional analysis (16–30 nt, 31–48 nt, and 49–50 nt). Overlaid pie charts show the proportions of aligned reads in each aforementioned sub-range that either had one unique best alignment (uniquely mapped) or multiple equally valid alignments (multimapped). **B** Proportions of alignments of uniquely mapped (top) and multimapping reads (bottom) from three sub-ranges (16–30 nt, 31–48 nt, and 49–50 nt) assigned to various annotated genomic features. Only features to which at least 1% of alignments were assigned are shown, with features to which less than 1% of alignments were assigned are represented in the 'other' category. 'Ambiguous' refers to an alignment that overlapped with more than one genomic feature and therefore cannot be assigned to a single feature

The vast majority of reads that aligned to the genome were observed in the range of 16–30nt, the range known to harbour miRNAs and piRNAs (Fig. 4A). However, neither of these classes made a substantial contribution to alignments of uniquely mapping or multimapping reads. Alignments to miRNA genes comprised approx. 0.3% of unique alignments and around 1% of alignments among multimappers. The most commonly assigned feature among 16–30nt reads was intron, comprising 32% of alignments of uniquely mapped reads and 27% of alignments of multimappers (Fig. 4B). The second most common feature in both cases was exon. Among alignments of longer reads (31–48 nt and 49–50 nt), the proportion of alignments assigned to features markedly dropped amongst uniquely mapping reads. The dominant feature assigned amongst longer reads was rRNA, with the exception of multimapping alignments of 31–48 nt reads, among which tRNA was the most common feature identified. Interestingly, miRNA genes were also represented amongst multimapping alignments of longer reads, suggesting that a fraction of reads derive from pre-miRNAs that have not yet undergone cleavage by Dicer.

#### (II) Differential expression of mRNA fragments derived from BOECs EVs

**Fig. 5:**
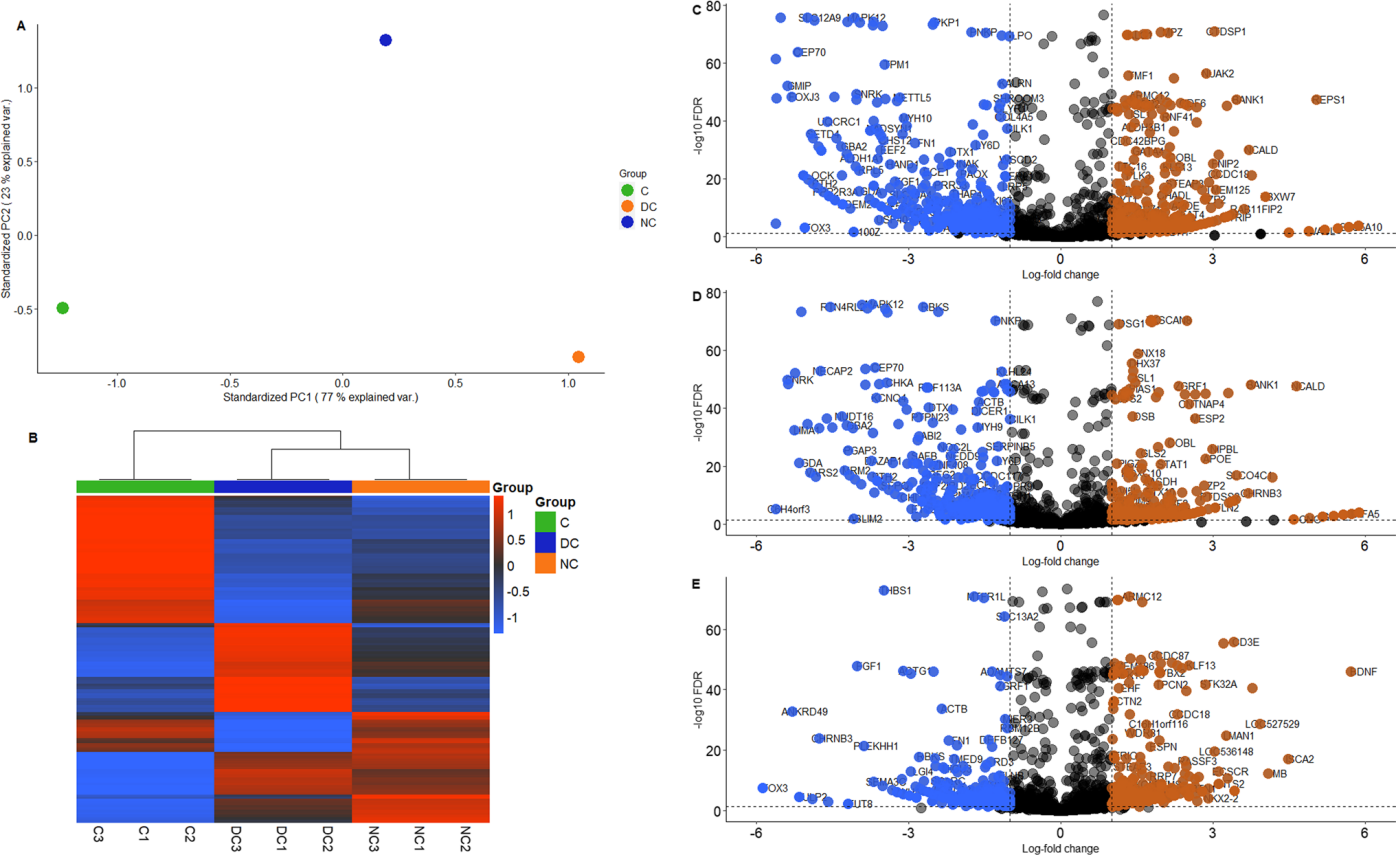
The profile of EV RNA fragments (16–48 nt) originating from exonic regions of protein coding RNA. **A** Principal component analysis (PCA) plot depicting inter and intra sample distances based on log2 fold changes in EV mRNA fragments derived from contact (green circle), direct contact (orange circle), non-contact (blue circle) co-culture of oviductal epithelial cells and spermatozoa. The samples in the same group are overlapping owing to the huge intergroup variation and less intragroup variation **B** Heatmap illustrating differential mRNA expression in co-culture models versus control. The blue shade denotes the depletion and the red shade denotes enrichment. **C** The contrast between the mRNA fragments derived from oviductal epithelial cell EVs in response to co-incubation with spermatozoa in a direct contact and non-contact versus control. In each plot the orange circles refer to enrichment and blue circles refer to significant depletion. **D** The volcano plot depicts the EVs mRNA fragments from non-contact co-culture of oviductal epithelial cells versus control. **E** This volcano plot demonstrates the contrast in mRNA fragments between direct contact co-culture EVs and EVs derived from non-contact co-culture of oviductal epithelial cells and spermatozoa

There was drastic differential enrichment in EV RNA fragments (16–48 nt) originating from exonic regions of protein coding RNA. The three groups (direct contact, non-contact, and control) showed very little intragroup variation while exhibiting very high intergroup variation in principal component analysis (Fig. 5 A and B). In direct-contact versus control comparison, there were 960 significantly enriched fragments and 917 significantly depleted fragments. 882 significantly enriched exonic mRNA fragments and 812 significantly depleted mRNA fragments were observed in non-contact vs control comparison. Between direct-contact and non-contact groups there were 878 significant enrichments and 1000 significant depletions. Only measurement of significance was FDR < 0.05 (Fig. 5C–E). mRNA fragments were considered enriched or depleted when they exhibited a log fold change of 1 or − 1 respectively.

**Fig. 6:**
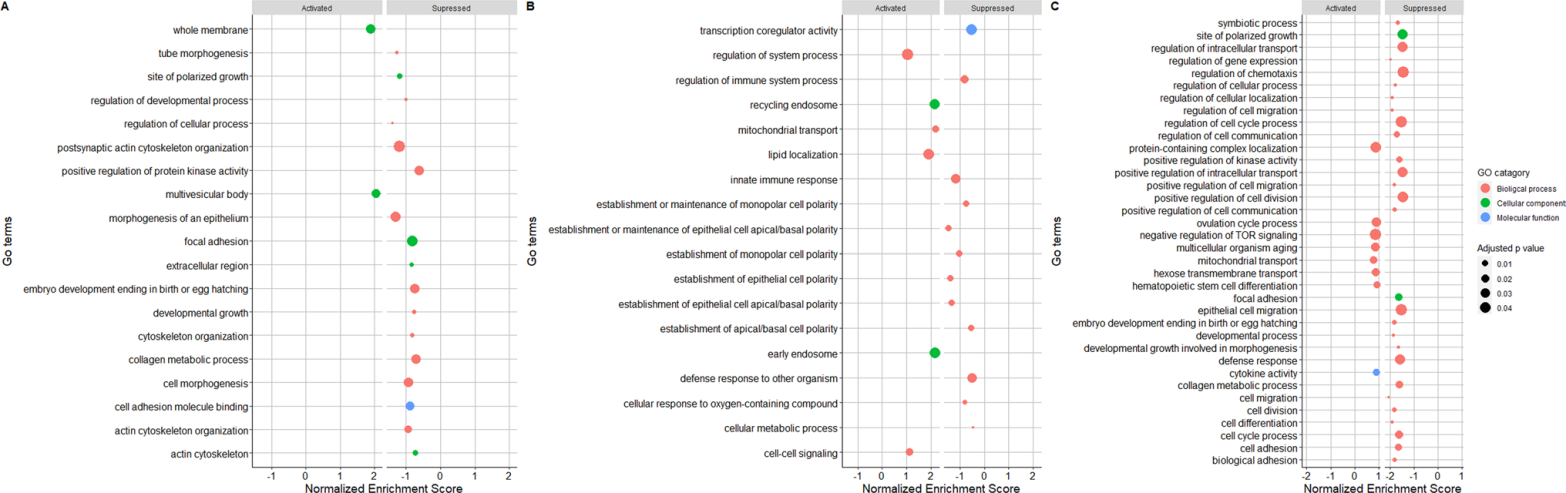
Biological pathways represented by EV derived exonic mRNA fragments **A** GO terms that are overrepresented in oviductal epithelial cells when spermatozoa is in direct contact **B** GO terms that are overrepresented in oviductal epithelial cells when the spermatozoa interacts remotely (non-contact co-culture). **C** GO terms that are overrepresented in oviductal epithelial cells direct versus non-contact co-cultures

The Gene set enrichment analysis (GSEA) of the differentially enriched mRNA fragments were performed based on the gene ontology (GO) annotations for *Bos Taurus*. GSEA revealed activation of microvesicular body associated pathways in contact cell culture versus control whereas most of the pathways were found to be suppressed (Fig. 6A). Some of the suppressed biological processes, molecular function and cellular components include focal adhesion, collagen metabolism, actin cytoskeleton organization and cytoskeleton protein binding. Interestingly the pathways linked with regulation and suppression of innate immune responses, defense against another organism were observed to be suppressed in case of non-contact co-culture versus control (Fig. 6B). The activated pathways in non-contact co-culture were lipid localization and endosome recycling as compared to control. Figure 6C illustrates pathways suppressed and activated in direct versus non-contact co-cultures.

#### (I) Differential expression of miRNA

**Fig. 7:**
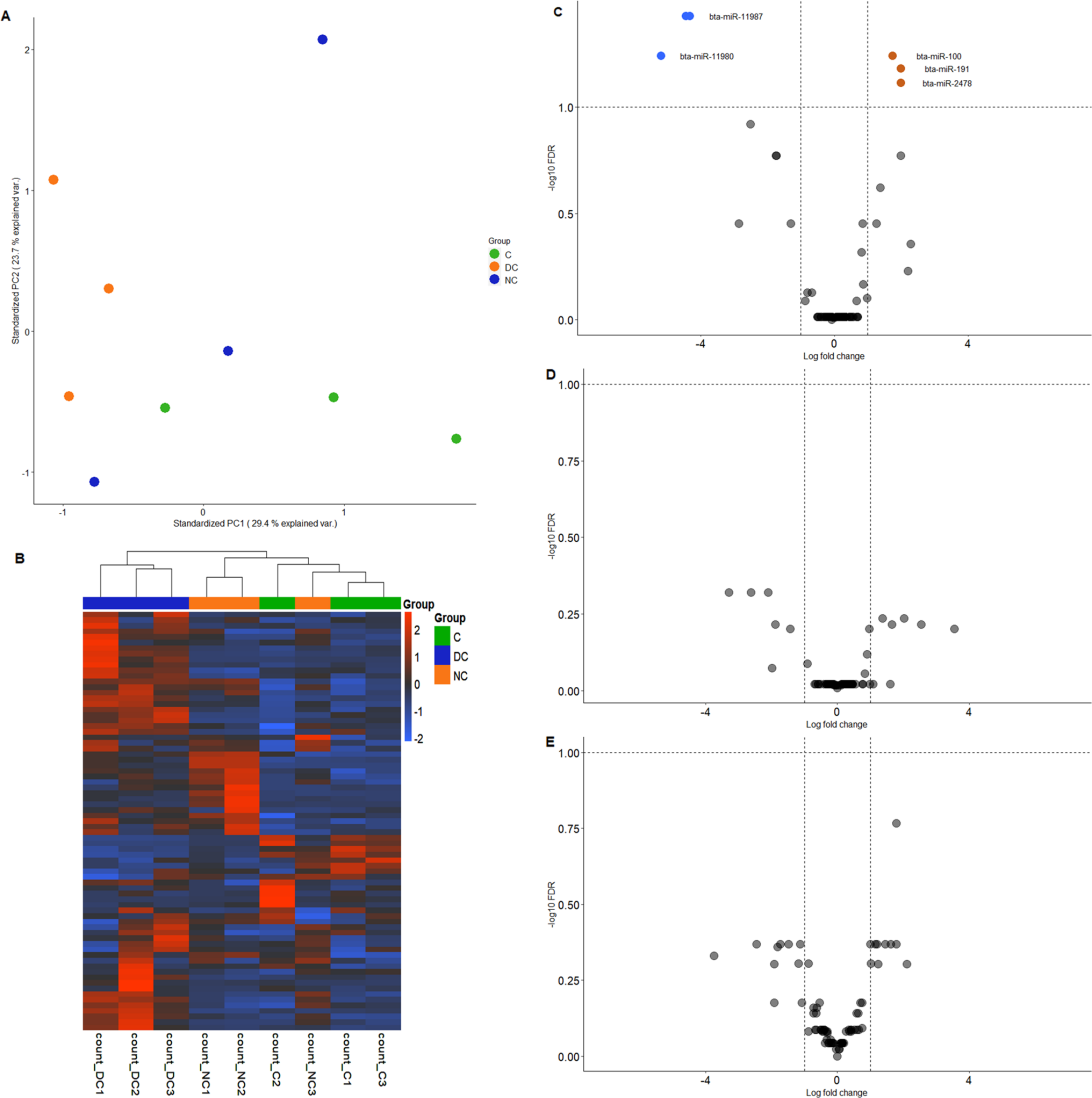
The miRNA profile of EVs derived from oviductal epithelial cells when incubated with spermatozoa in a direct and non contact co-culture models. **A** Principal component analysis (PCA) plot depicting inter and intra sample distances based on log2 fold changes in EVs miRNAs derived from contact (green circle), direct contact (orange circle), non-contact (blue circle) co-culture of oviductal epithelial cells and spermatozoa. **B** Heatmap illustrating differential miRNA expression in co-culture models versus control. The blue shade denotes the depletion, and the red shade denotes enriched miRNAs. The orange-colored circles refer to enrichment and blue coloured circles refer to significant depletion **C** The volcano plot representing the distinct miRNAs carried by EVs from direct contact co-culture of oviductal epithelial cells and spermatozoa versus control **D** Volcano Plot demontrating no significant differences in miRNAs obtained from non-contact co-culture versus control **E** This volcano plot demonstrates the contrast in miRNAs between direct contact co-culture EVs and EVs derived from non-contact co-culture of oviductal epithelial cells and spermatozoa. miRNAs were considered significantly enriched or depleted when they expressed a log fold change of either 1 or − 1 respectively and a FDR of less than 0.1

The sequencing results revealed that the miRNAs carried by EVs in contact co-culture varies from the control group. The PCA plot (Fig. 7A) represents the segregation of the samples within different groups. Three miRNAs (bta-miR-100, bta-miR-191, bta-miR-2478) were significantly enriched and two miRNAs were significantly depleted (bta-miR-11987, bta-miR-11980) in direct-contact co-culture group compared to control. We did not obtain any significantly differentially expressed miRNAs in non-contact co-culture group versus control (Fig. 7B–E). Due to the high intragroup variation, a FDR less than 0.1 was considered to determine the statistical significance instead of the more commonly used 0.05 threshold.

**Fig. 8:**
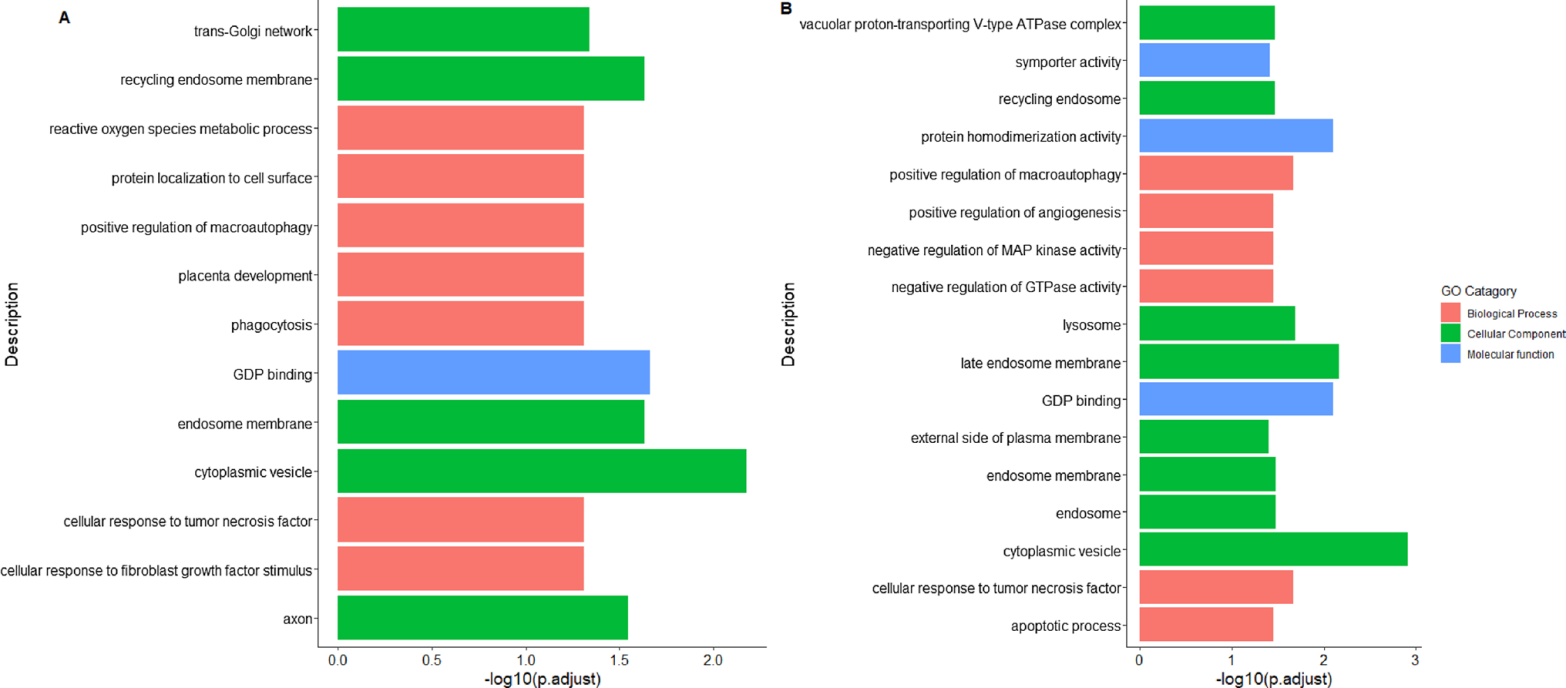
The GO pathway enrichment analysis **A** The plot represents the GO terms that are overrepresented amongst targets of differentially expressed EV miRNAs when spermatozoa are in direct contact with the oviductal epithelial cells **B** The plot represents the GO terms that are overrepresented amongst targets of differentially expressed EV miRNAs in non-contact versus direct contact co-cultures

In addition, we performed GSEA based on GO of target genes associated with enriched miRNAs. There was only one pathway enriched in non-contact versus control which was associated with inner cell mass proliferation. The biological processes, molecular function and cellular components of pathways linked with direct contact versus control and direct contact versus non-contact are depicted in figure (Fig. 8A, B).

## Discussion

Previous studies have established the importance of EVs in mediating cell–cell communication in diverse events associated with reproduction including attainment of functional maturation by spermatozoa, fertilization, implantation, and maintenance of pregnancy (Hasan et al. 2021; Li and Winuthayanon 2017). Investigations have reported the role of oviductal extracellular vesicles in enhancing the fertilizing ability of spermatozoa (Al-Dossary et al. 2013; Bathala et al. 2018), improving embryo development and cryoresistance (Almiñana et al. 2017; Lopera-Vásquez et al. 2016) and increasing the efficacy of embryo transfer by increasing the birth rate (Qu et al. 2019). The presence of spermatozoa influencing the transcriptomic profile of the oviduct is very well documented, but the impact of spermatozoa on EVs derived from oviductal extracellular vesicles is not much studied. A recent study by our group has demonstrated that bio-active particles released from spermatozoa caused changes in the transcriptomic profile of the oviductal epithelial cells in cattle (Reshi et al. 2020) implying a remote cross-talk between oviduct and spermatozoa. The current study sought to determine the changes in the EVs production and RNA cargo of EVs derived from bovine oviductal epithelial cells when incubated with spermatozoa in a direct and non-contact co-culture model. Our results suggested that there is a rise in the number of EVs released from oviductal epithelial cells, thus it seems oviductal cells are responding to the presence of spermatozoa in co-culture by increasing the production/release of EVs to the periconception environment. In addition, distinct cargoes in form of miRNAs were present in the EVs derived from BOECs in response to spermatozoa. Surprisingly, the differences were observed in cargo obtained from BOECs EVs from direct contact versus non-contact co-culture of spermatozoa and BOECs. This implies that spermatozoa non-contact and direct contact with oviductal epithelial cells stimulates different reactions which impacts the release of specific cargo via EVs.

As already mentioned transcriptomic profile of oviductal epithelial cells is altered while interacting with spermatozoa (Almiñana et al. 2014; Fazeli et al. 2004; Kodithuwakku et al. 2007; Li et al. 2010; Reshi et al. 2020) but very little is known concerning whether the release of EVs is affected after sperm arrival in the oviduct. The NTA analysis from our data suggests a significant rise in the number of EVs in co-culture models of oviductal epithelium and spermatozoa when compared to control. This implies that the oviductal epithelium responds to arrival of spermatozoa by increasing the production of EVs. Although the number of EVs were higher in contact co-culture, the striking observation was that spermatozoa without having a direct contact with oviductal cells, still increased the number of EVs production/release from the oviductal cells. The increase in the number of EVs in co-culture models can be linked with specific cargo being packed in them versus the EVs derived from control.

Recent reports suggest that miRNAs, in addition to acting inside the cells, can be even released by cells via EVs into the extracellular matrix facilitating vital cell signalling events as well as other aspects of intercellular communication in normal as well as pathophysiological events (Liang et al. 2017). In case of direct contact versus control, the GSEA with *Bos taurus* GO term annotations revealed the role of the miRNAs in the biological processes associated with phagocytosis, positive regulation of macro autophagy, cellular responses to tumour necrosis factor (TNF) and fibroblast growth factor (FGF), placenta development. A study has demonstrated that the autophagy pathways remain active throughout the early embryogenesis in humans and play essential role in its development (Song et al. 2022). Furthermore, another study conducted in mice revealed that post fertilization macroautophagic is immediately activated and is important for the development of pre implanted embryo (Tsukamoto et al. 2008; Yamamoto et al. 2014). Moreover, another study revealed the localization of FGF in rat oviduct during early pregnancy (Alan and Liman 2021). During the process of embryonic development FGFs serve a crucial role by regulating cellular proliferation, differentiation, and migration (Cotton et al. 2008). A study in bovine shows the spermatozoa on interacting with oviductal epithelial cells regulates TNF expression and phagocytosis, which aids its survival in the oviduct (Yousef et al. 2016). In addition, the miRNAs in the case of non-contact versus control were also found to regulate pathways associated with inner cell mass proliferation. The collective notion from these results suggests that spermatozoa not only support its own survival but stimulates oviduct to pass information regarding the aforementioned processes onto early embryo via oviductal EVs. While only one spermatozoon is required to fertilize the oocyte, it seems the presence of other spermatozoa is required to establish a suitable environment for the semi allogenic embryo to survive. This also reflects the existence of a symbiotic association between spermatozoa and the oviduct, where the oviduct supports the viability of spermatozoa and the later prepares the oviduct for the arrival of the embryo (Li and Winuthayanon 2017). Additionally, studies report that epigenetic reprogramming of the early embryo occurs in the oviduct (Ferraz et al. 2018; Pérez-Cerezales et al. 2018). As already mentioned EVs are uptaken by the embryo and this eventually leads to alteration in the embryonic transcriptome (Bauersachs et al. 2020) and based on our results the collective notion suggests that spermatozoa induce the oviduct to produce EVs that act as the main players of the cross talk between oviduct-spermatozoa-early embryo.

Due to the nature of the library preparation, which was focused on the small RNA, we cannot expect to detect full length mRNA in the sequencing data. A significant number of reads aligned to the protein coding genes. However, the majority of these mapped reads were from 16 to 48 bases in length implying a rather fragmented population of “EV derived mRNA”. The phenomenon of fragmented RNA in EVs is not a novel one. There have been multiple reports on the level of fragmentation in EV RNA and their possible functionality (O’Brien et al. 2020; Prieto-Vila et al. 2021). Assuming that EV derived RNA is representative of the transcriptome of the cells that produce the EVs, in this case oviductal epithelium, we hypothesize that the EV RNA can be used as an analogue for the cellular transcriptome. Even in the highly fragmented state, EV derived RNA can be useful to convey a “snapshot” of the EV source’s transcriptome and the physiological state. Thus, we have conducted differential enrichment analysis and network analysis on EV RNA reads that uniquely mapped to the exonic regions of the bovine transcriptome. Only the EV RNA reads that uniquely mapped to the exonic regions were selected for the analysis to increase the accuracy of transcriptomic representation.

In the case of the non-contact co-culture the mRNA fragments found in EVs were linked with the genes that were mostly involved in suppressing and regulating the immune system. Several studies in cattle have found that spermatozoa-oviduct binding generates anti-inflammatory responses that favour spermatozoa survival (Marey et al. 2016; Talukder et al. 2020). Therefore, our present study provides vital insights regarding the role that EVs might play in such immune system activation during sperm-oviduct interactions. Some studies also revealed a comparable system at work in the female reproductive tract to protect the embryo. The semi-allogenic embryo, to avoid the maternal immune system, releases biomolecules that decrease the inflammation in the bovine oviduct (Maillo et al. 2015; Talukder et al. 2018). The spermatozoa retains its motility for up to 3–4 days in the mammalian oviduct, and this timeline also overlaps with the presence of the early embryo in the oviduct (Camara Pirez et al. 2020; Li and Winuthayanon 2017). Hence there is a possibility that suppression of the immune system is a repercussion of synergistic effects of the early embryo and spermatozoa stimulating the oviduct to release EVs containing cargoes that mediate the processes related to immune responses. These findings point to the possibility that in order to evade the maternal immune attack, the suppression of immunity takes place before spermatozoa establish the direct contact with oviductal epithelial cells.

The gene set enrichment analysis revealed certain pathways that were suppressed or activated in both the co-culture models. The pathway enrichment analysis revealed that EV mRNA from direct co-culture was associated with genes that were involved in the activation of multivesicular body (MVB) pathway. Interestingly the suppressed pathways included focal adhesion and collagen metabolism. The requirement for successful implantation is attachment of the embryo to the endometrium and adhesion molecules are necessary to initiate this process. A series of cytoskeletal reorganization events and expression of adhesion molecules takes place in the endometrium to initiate the process of attachment to the embryo (Achache and Revel 2006). The possible explanation of this pathway being suppressed in the oviduct can be avoiding the occurrence of ectopic pregnancy as the oviduct is not the site where the embryo has to attach. Furthermore, the cell adhesion molecule binding pathway and the actin cytoskeleton organization pathway are suppressed which are essential for embryo attachment (Madawala et al. 2016; Singh and Aplin 2009). It can therefore be assumed that spermatozoa interact with the oviduct and induces it to suppress the collagen metabolism pathways. This plays a pivotal role in remodelling extracellular matrix in the endometrium during pregnancy (Shi et al. 2020). This also can be an explanation for extremely rare incidences of ectopic pregnancies in cows (Corpa 2006). Hence, it is reasonable to speculate an increase in the EV numbers in the oviduct occurs after the arrival of spermatozoa, because spermatozoa stimulate the oviduct to a physiological state that is conducive to embryo development, but not oviductal implantation.

We showed that the small RNA composition of BOEC EVs was variable and depended on read length, with a high proportion of the RNA comprising intron-derived transcripts. While it is likely that many such intronic transcripts comprise transcriptional waste, intron-derived transcripts may also comprise functional noncoding RNA. Indeed, a high proportion of human miRNAs are produced from introns (Steiman-Shimony et al. 2018), as are most small nucleolar RNAs (snoRNA) (Hubé et al. 2017). It is therefore possible that some intron-derived transcripts found within the BOEC EVs comprise novel small RNAs which are not yet annotated. The abundances of these intron-derived transcripts differed sufficiently between treatment groups to result in differential expression of their host genes. While such differential expression does not necessarily indicate a functional role of these transcripts, it nevertheless betray information about the physiological state of the cells that produced them. Such material, even if it has no functional role in recipient cells, may therefore comprise a rich pool of biomarkers. Furthermore, we acknowledge the fact that that spermatozoa retains some bioactive particles post percoll washing which also might influence the results obtained. However, the number of these nanoparticles is of miniscule amounts and their RNA cargo is below the detection limit. Moreover, this study gives a general idea regarding the physiological state of the BOECs based on BOEC derived EVs in response to spermatozoa. However, the downstream effects of these EVs on embryo, spermatozoa and oviduct has not been addressed experimentally by this study which therefore can be taken into consideration for further experiments. In addition, we would like to emphasize that the purification protocol used to isolate EVs allows for EV enrichment and mostly get rid of the contaminants. Although we did get rid of the contaminants from the EVs but not completely as to best of our knowledge, currently, no EV purification system is effective at removing contaminants completely. Hence the enriched EV elute analysed in this study have retained some of the contaminants and might have influenced the results.

It also would be intriguing to study if the spermatozoa from bulls with low fertility index would be able to generate these responses from the oviduct. Several scientists in the past have argued that the function of oviduct and spermatozoa is not confined to just fertilization, yet a precise oviduct-spermatozoa dialogue is required for the successful pregnancy (Li and Winuthayanon 2017; Pérez-Cerezales et al. 2018). Our present data also implies that male gametes extensively alter and prime the female reproductive tract and these interactions might take part in pre and post-fertilization events aiding a successful implantation process as well as for supporting embryo quality. Our findings could add valuable insights for a better understanding of the potential role that EVs could play as modulators as well as facilitators of sperm-oviduct and embryo-maternal interactions and their implications for better fertility treatments in the future in modulating infertility issues faced by mankind.

## Conclusion

In conclusion, our results suggested that spermatozoa through contact or remotely, alter the cargo of EV secreted by female reproductive tract epithelial cells. Therefore, the oviduct is not merely a passageway for spermatozoa or the site of fertilization but a dynamic organ where gametes and oviduct communicate in a well-orchestrated series of events leading to the initiation of new life. Over the years, awareness regarding the pre-conception environment has remained limited as the role of oviduct in the pregnancy establishment has been under-investigated. The interaction between spermatozoa and oviduct during pre-conception is the epoch-making step that determines the fate of early embryo development. EVs could be the missing link that play an essential role in mediating the communication during these events at early stages of pregnancy and offspring production.

## Supplementary Information

Below is the link to the electronic supplementary material.

Supplementary file1 (DOCX 20 KB)

Supplementary file2 (XLSX 341 KB)

## Data Availability

The sequencing data has been uploaded to NCBI SRA repository (www.ncbi.nlm.nih.gov/sra) under the accession no. PRJNA842680. The proteomics data has been uploaded in the PRIDE (Proteomics Identification Database) repository under the accession number PXD038106.

## Abbreviations

BOECs: Bovine oviductal epithelial cells
DE: Differential expression
EVs: Extracellular vesicles
FDR: False discovery rate
GSEA: Gene set enrichment analysis
NTA: Nanoparticle tracking analysis
PCA: Principal component analysis
RNAseq: RNA sequencing
TEM: Transmission electron microscopy
