# Supplementary material for: Spermatozoa, acts as an external cue and alters the cargo and production of the extracellular vesicles derived from oviductal epithelial cells in vitro
Source: J Cell Commun Signal. 2022 Dec 5;17(3):737–55. doi: 10.1007/s12079-022-00715-w (PMC10409707; doi:10.1007/s12079-022-00715-w)
Supplement: Supplementary file 1 — Supplementary file1 (DOCX 20 KB) [file 12079_2022_715_MOESM1_ESM.docx]

| **Table SI:** Intensity values of selected proteins in BOEC neat and BOEC EV purified samples* | | |
| --- | --- | --- |
| **Protein names** | **Intensity BOECs EVs- Neat** | **Intensity BOECs EVs-Purified** |
| A2M | 2.22E+08 | 9.85E+08 |
| ACTB | 0.00E+00 | 8.61E+06 |
| ACTN1 | 0.00E+00 | 2.12E+06 |
| ACTN4 | 0.00E+00 | 1.81E+07 |
| AHCY | 0.00E+00 | 2.93E+06 |
| ALB | 1.13E+12 | 1.08E+11 |
| ALDOA | 2.72E+05 | 3.21E+08 |
| ANXA1 | 5.68E+06 | 1.07E+09 |
| ANXA11 | 0.00E+00 | 1.51E+07 |
| ANXA2 | 3.35E+07 | 1.53E+09 |
| ANXA5 | 0.00E+00 | 2.18E+08 |
| ANXA6 | 0.00E+00 | 6.20E+07 |
| ANXA7 | 0.00E+00 | 3.69E+07 |
| ATP1A1 | 4.80E+05 | 1.78E+08 |
| BSG | 0.00E+00 | 6.84E+07 |
| C3 | 1.57E+07 | 2.12E+06 |
| CCT2 | 0.00E+00 | 5.15E+07 |
| CCT3 | 0.00E+00 | 2.57E+07 |
| CCT4 | 0.00E+00 | 2.90E+07 |
| CCT6A | 0.00E+00 | 1.63E+07 |
| CCT8 | 0.00E+00 | 2.47E+07 |
| CD81 | 0.00E+00 | 1.24E+07 |
| CD9 | 0.00E+00 | 2.16E+08 |
| CDC42 | 0.00E+00 | 1.93E+07 |
| CFL1 | 5.39E+05 | 6.10E+07 |
| CLTC | 0.00E+00 | 2.65E+07 |
| EEF1A1 | 0.00E+00 | 1.21E+08 |
| EEF2 | 0.00E+00 | 1.12E+07 |
| ENO1 | 4.97E+06 | 1.43E+08 |
| EZR | 7.23E+06 | 7.13E+08 |
| FLNA | 0.00E+00 | 4.86E+06 |
| FLOT1 | 0.00E+00 | 1.23E+06 |
| FN1 | 4.57E+06 | 5.95E+07 |
| GAPDH | 6.61E+06 | 1.70E+08 |
| GDI2 | 0.00E+00 | 2.49E+07 |
| GNAI2 | 0.00E+00 | 1.12E+08 |
| GNAS | 0.00E+00 | 6.51E+06 |
| GNB1 | 0.00E+00 | 1.26E+07 |
| GNB2 | 2.88E+05 | 1.07E+08 |
| GPI | 0.00E+00 | 1.61E+08 |
| HSP90AA1 | 0.00E+00 | 1.04E+08 |
| HSP90AB1 | 2.19E+06 | 4.78E+07 |
| HSPA8 | 1.23E+07 | 1.56E+08 |
| IQGAP1 | 0.00E+00 | 2.24E+07 |
| ITGB1 | 1.09E+06 | 1.51E+08 |
| KRT1 | 4.78E+07 | 6.10E+09 |
| KRT10 | 4.75E+07 | 7.51E+09 |
| LDHA | 1.18E+05 | 5.81E+07 |
| LDHB | 0.00E+00 | 1.93E+07 |
| LGALS3BP | 0.00E+00 | 6.57E+07 |
| MSN | 0.00E+00 | 6.37E+07 |
| MYH9 | 0.00E+00 | 3.66E+07 |
| PDCD6IP | 0.00E+00 | 8.81E+07 |
| PFN1 | 0.00E+00 | 1.90E+07 |
| PGK1 | 8.51E+05 | 9.95E+07 |
| PKM | 2.79E+06 | 1.38E+08 |
| PPIA | 2.10E+06 | 8.30E+07 |
| PRDX1 | 0.00E+00 | 2.10E+07 |
| RAB5C | 0.00E+00 | 9.52E+06 |
| RAB7A | 0.00E+00 | 2.07E+07 |
| RAC1 | 0.00E+00 | 3.01E+07 |
| RALA | 0.00E+00 | 3.20E+07 |
| RHOA | 0.00E+00 | 8.42E+07 |
| SDCBP | 0.00E+00 | 8.41E+07 |
| SLC3A2 | 0.00E+00 | 1.66E+07 |
| TCP1 | 0.00E+00 | 4.74E+07 |
| TFRC | 0.00E+00 | 9.23E+07 |
| TPI1 | 0.00E+00 | 5.27E+07 |
| TSG101 | 0.00E+00 | 4.72E+06 |
| TUBB4B | 7.12E+06 | 2.18E+08 |
| VCP | 3.01E+07 | 2.37E+07 |
| YWHAB | 0.00E+00 | 7.07E+06 |
| YWHAE | 0.00E+00 | 1.19E+07 |
| YWHAG | 0.00E+00 | 2.23E+07 |
| YWHAQ | 0.00E+00 | 6.02E+06 |
| YWHAZ | 0.00E+00 | 1.96E+08 |
| *Proteins reported in the table were selected from the top 100 most reported proteins in the Vesiclepedia database; BOEC- bovine oviductal epithelial cells; EV- extracellular vesicles | | |
